# Combination of mangiferin and T0901317 targeting autophagy promotes cholesterol efflux from macrophage foam cell in atherosclerosis

**DOI:** 10.1186/s13020-023-00876-9

**Published:** 2024-01-05

**Authors:** Qian Chen, Sijian Wang, Ruixia Bao, Dan Wang, Yuzheng Wu, Yi Zhang, Mengyang Liu, Tao Wang

**Affiliations:** 1 State Key Laboratory of Component Based Chinese Medicine, Institute of Traditional Chinese Medicine, Tianjin University of Traditional Chinese Medicine, 10 Poyanghu Road, Tianjin, 301617 China; 2 State Key Laboratory of Bioactive Substance and Function of Natural Medicines, Institute of Materia Medica, Chinese Academy of Medical Sciences and Peking Union Medical College, 1 Xian Nong Tan Street, Beijing, 100050 China

**Keywords:** Mangiferin, T0901317, Cholesterol efflux, Autophagy, Macrophage foam cells

## Abstract

**Background:**

The synthetic liver X receptor ligand (LXR) T0901317 (T0) has been reported to attenuate atherosclerosis (AS) without hyperglyceridemia due to innovative drug combination or nano-sized drug delivery. Given the key roles of mangiferin (MGF) in lipid metabolism and atherogenesis, it is critical to investigate progression of atherosclerotic lesion after combined treatment of MGF and T0.

**Methods:**

Atherosclerotic plaque formation and hepatic lipid accumulation were compared in *Apoe*^*−/−*^ mice among T0 and/or MGF treatment. The in vitro functions of MGF and T0 were analyzed by Oil-red O staining, cholesterol efflux assay, transmission electron microscopy and western blot analyses with or without acetylated low density lipoprotein.

**Results:**

The combination therapy are effective regulators for atherosclerotic plaque formation in *Apoe*^*−/−*^ mice, due to upregulation of ABCA1 and ABCG1 induced by LXR activation. Subsequently, we identified autophagy promoted by MGF and T0 treatment establishes a positive feedback loop that increases cholesterol efflux, resulted from LXRα activation. Under atherogenic conditions, the autophagy inhibitor CQ abolished the enhancement effect on cholesterol outflow of MGF and T0. Mechanically, MGF and T0 promotes LXRα and mTOR/AMPK signaling cascade in macrophage, and promotes AMPK signaling cascade in hepatocyte, leading to lipid metabolic homeostasis.

**Conclusions:**

Altogether, our findings reveal that MGF and T0 engages in AS therapy without side effects by activating AMPK-dependent autophagy to promote macrophage cholesterol efflux, and MGF might serve as a natural compound to assist T0 in AS via targeting autophagy.

**Supplementary Information:**

The online version contains supplementary material available at 10.1186/s13020-023-00876-9.

## Introduction

Atherosclerosis (AS) is the major underlying risk of cardiovascular outcomes with serious mortality worldwide [1]. It is well established that the formation of macrophage foam cells is the initial cellular event of atherosclerotic lesions [2]. Clearance of the arterial cholesterol deposition by macrophage is beneficial during the early stages of AS [3], however, as the overwhelmed cholesterol disrupts the cellular feedback regulation, the macrophages accumulating lipid droplets become foam cells that contributes to AS progression [4]. Disturbed cholesterol-modulating machinery in macrophages, especially impaired cholesterol efflux ability, correlates closely with formation of foam cell and disruption of reverse cholesterol transport. Consequently, it is an attractive antiatherogenic strategy to enhance cholesterol efflux from macrophage foam cells to the liver for the cholesterol clearance into the bile and ultimately the feces [5]. Accordingly ATP-binding cassette transporters from subfamilies A1 and G1 (ABCA1 and ABCG1) have been identified as being crucial for this process [6]. Strategies to enhance the cholesterol efflux by increasing the expression of ABCA1 and ABCG1, which effectively alleviated excess cholesterol accumulation in the macrophages to acquire hypolipidemic effects in the progression of atherosclerotic lesions.

Autophagy, a double membrane-driven process of lysosomal degradation and recycling of cellular components, is critically involved in the development of early and advanced atherosclerotic plaques formation [7]. Under atherogenic conditions, macrophage lipophagy promotes the intracellular breakdown of lipids and the delivery of hydrolyzed cholesterol esters to lysosomes for ATP-binding cassette transporters mediated cholesterol efflux in foam cells [8]. Therefore, autophagy stimulation contributes to maintaining cellular lipid homeostasis in the process of atherosclerotic plaque formation.

Synthetic liver X receptor (LXR) agonists, such as T0901317 (T0) and GW3965, are effective regulators for AS therapy in animal models and clinical trials, due to upregulating expression of ABCA1 and ABCG1 [9]. LXR has been proven to inhibit foam cell formation by LXR-induced upregulation ABCA1 and ABCG1, which efficiently induces the cholesterol efflux. Macrophage overexpression of ABCA1 and ABCG1 accelerates reverse cholesterol transport and then greatly alleviate macrophage dysfunction and atherogenesis [10]. Although LXR agonists were advanced to clinical development for the treatment of AS, unfortunately, in the liver, LXR agonists activate lipogenesis by upregulation of sterol regulatory element binding transcription factor 1c (SREBP-1c), and thereby substantially induce fatty liver and hyperglyceridemia [11].

Mangiferin (MGF), derived from *Mangifera indica* L. (Anacardiaceae), is a natural C-glucosyl xanthone and polyhydroxy polyphenol, which has been well reported for its hypoglycemic activity and hypolipidemic activity [12, 13]. Our previous study has shown that MGF significantly decreased liver triglyceride (TG) and free fatty acid (FFA) levels mainly depending on sirtuin-1 (SIRT-1)/ AMP-activated protein kinase (AMPK) activation to reduce hepatic lipid synthesis, promote lipolysis and fatty acid oxidation [14]. Furthermore, another study exhibited that in overweight patients with hyperlipidemia, participants receiving MGF showed decreased serum TG and FFA levels and insulin resistance index with increased plasma high-density lipoprotein cholesterol level and lipoprotein lipase activity [15]. Additionally, the hypolipidemic effects of MGF was also confirmed to promote cholesterol efflux from macrophages through PPARγ-LXRα-ABCA1/G1 pathway in atheromatous plaque formation in *Apoe*^*−/−*^ mice [16]. Thus, these results suggest that co-treatment of MGF and T0 could be a good strategy to prevent AS related dyslipidemia targeting autophagy in cholesterol efflux from macrophage foam cells.

Actually the synthetic LXR ligand T0 has been widely reported to reduce atherosclerosis but activated lipogenesis. Although literatures and our previous study has shown that MGF inhibits hepatic steatosis, it has limited cardioprotective actions. In this paper, we performed combined use of MGF and T0 in *Apoe*^*−/−*^ mice and macrophages in vitro to elucidate whether the combined treatment exerts hypolipidemic effects in atherosclerotic plaque formation without fatty liver and hyperglyceridemia.

## Materials and methods

### Materials

Primary rabbit polyclonal antibodies against AMPK (2532), *p*-AMPK (2535), mTOR (2983), *p*-mTOR (39182), ACC (3676), *p*-ACC (11818), SCD-1 (2794S), ATGL (2439S), CPT-1 (97361S), and goat anti-rabbit IgG (whole molecule) were purchased from Cell signaling technology, Inc. (Danvers, MA, USA). Primary rabbit polyclonal antibodies against ABCA1 (ab18180), ABCG1 (ab218528), LC3 (ab192890), SREBP-1c (ab28481), FAS (ab133619), and β-actin (ab8227) were purchased from Abcam (Cambridge, MA, USA). Primary rabbit polyclonal antibodies against α-SMA (14395), CD36 (18836), Beclin1 (11306), ATG5 (10181), and ATG7 (10088) were purchased from Proteintech Group, Inc. (Rosemont, IL, USA). TG and total cholesterol (TC) assay kits were purchased from BioSino Bio-Technology and Science Inc. (Beijing, China). T0 and MGF were purchased from MedChemExpress, Int. (Shanghai, China).

### Cell culture

RAW264.7 cells (a murine macrophage cell line) and THP-1 cells (a human monocytic cell line) were purchased from ATCC (Rockville, MD, USA) and cultured in DMEM or PRMI 1640 medium containing 10% FBS, 1% penicillin/streptomycin and 2 mmol L^−1^ glutamine.

### Primary peritoneal macrophage culture

Peritoneal macrophages were collected from peritoneal cavity at third day after male C57/BL6J mice (aged 8 weeks) were injected with 4% thioglycolate broth as described previously [17]. Then the cells were washed with phosphate-buffered saline, centrifuged at 400 g for 5 min, and suspended in DMEM containing 10% heat-activated FBS, 1% penicillin/streptomycin and 2 mmol L^−1^ glutamine.

### Animals

*Apoe*^*−/−*^ mice (18–22 g, male, 8 weeks old) with C57/BL6J background were purchased from Beijing Vital River Laboratory Animal Technology (China). All mice were maintained under a 12:12 h light/dark cycle, at 23 ± 1 ℃ with a relative humidity of 40–70% before and throughout the experiment. The mice had free access to water and a high-fat diet (HFD, 21% fat plus 0.5% cholesterol) during the treatment. After 1 week adaption, experimental mice were randomly allocated into four groups (10 per group) and received the following treatment for continuous 16 weeks: Ctrl, vehicle; T0, oral gavage of T0 (1 mg kg^−1^ bodyweight/day); MGF, oral gavage of MGF (200 mg kg^−1^ bodyweight/day); T0 + MGF, oral gavage of T0 and MGF. The dosages of MGF and T0, and the drug ratio and dosage of MGF and T0 combination were determined by previous studies [14, 18]. During the experiment, the mice were checked daily for bodyweight and intake of food and water. At the end of the experiment, all mice were sacrificed by an overdose of 2,2,2-tribromoethanol (640 mg kg^−1^, i.p. injection), followed by collection of aorta, liver and blood samples.

### Atherosclerotic lesion analysis

The aortas were obtained and used to determine *en face* lesions or prepare 5 μm frozen sections of aortic root cross sections for sinus lesion area quantification using Oil-red O staining. The lesion areas in *en face* aorta were calculated using the AxioVision program. For analyzing aortic root sinus plaque lesions, cryosectioning was performed. The digital images were photographed using an Axio Imager D2 (Zeiss, Oberkochen, Germany). Collagen content of aortic root cross section was determined using Masson staining.

### Immunofluorescence assay

Sections of aortic root or liver from mice and cells samples were blocked in 1% BSA containing 1% goat serum for 60 min. The slides were incubated with primary antibodies diluted by 1% goat serum overnight at 4 ℃. After incubation with Alexa-Fluor 555-conjugated goat anti-rabbit IgG antibody for 1 h at room temperature. Meanwhile, nuclei were stained with DAPI (blue) and then observed with an inverted fluorescent microscope (Carl Zeiss, Oberkochen, Germany). Calcification and expression of smooth muscle α-actin (α-SMA), ABCA1, ABCG1 and LC3 protein (red) in lesion areas or liver sections were determined by immunofluorescent staining with aortic root cross section or liver section. The colocalization of GFP-MAP1LC3B/LC3B (green) and RFP-LC3B (red) or bodipy (green) and LAMP2 (red) in AcLDL-treated peritoneal macrophages were analyzed by immunofluorescent staining.

### Serum and hepatic lipid analysis

Blood samples and liver tissues were collected to determine levels of TG, TC, FFA, AST and ALT by commercial analysis kits as previously described [19]. Liver tissue was also removed and used to prepare frozen or paraffin sections following the standard procedure. The paraffin sections were used for histopathological examination by hematoxylin–eosin (HE) staining. The frozen sections were used to assess lipid content by Oil-red O staining. The morphological changes were viewed under the light microscope (400 × amplification; BX43, Olympus, Japan).

### Determination of foam cell formation

Primary peritoneal macrophages were collected and plated on cover slips in 24-well plates. After attachment for 2 days, cells in serum-free PRMI 1640 medium were incubated with acetylated low density lipoprotein (AcLDL) for 5 h to induce foam cell formation, followed by treatment of T0, MGF, or T0 + MGF for 16 h. Cellular cholesterol content was determined using TC assay kit.

Primary peritoneal macrophages were treated with AcLDL for 5 h and different treatments for 16 h as described above. The attached cells were fixed and conducted Oil-red O staining to measure foam cell formation. In addition, cellular calcification and expression of bodipy, 4’,6-duamidino-2-phenylindole (DAPI), ABCA1, ABCG1, LAMP2, LC3 protein in vitro were determined by immunofluorescent staining.

### Cholesterol efflux assay

Primary peritoneal macrophages and THP-1 cells were collected and plated in black 96-well plates. Cells were treated with AcLDL for 5 h as described above. Then cells were incubated with medium containing T0, MGF, or T0 + MGF with both ApoA-1 (50 μg mL^−1^) and HDL (20 μg mL^−1^), with or without chloroquine (CQ) for 16 h. Cholesterol efflux assay kit obtained from Abcam (Cambridge, MA, USA) was used to detect the effects of different treatments on cholesterol outflow rate following the instructions.

### Transmission electron microscopy

The cell samples for transmission electron microscopy (TEM) analysis were fixed in 2.5% glutaraldehyde prior to post-fixation in osmium tetroxide and uranyl acetate en bloc staining. Samples were processed and embedded in Spurr epoxy resin, ultrathin sectioned (50 nm), and counterstained with lead citrate. Sections were analyzed using a Hitachi H-7650 TEM (Hitachi, Tokyo, Japan).

### Western blot

Total cellular proteins were extracted from cells or liver samples. The protein analysis by western blot was as described previously [20]. Protein bands were detected and analyzed using a ChemiDoc^™^ MP Imaging System (Bio-Rad Laboratories, CA, USA). β-actin was used as a loading control. Results were expressed as the integrated optical density relative to β-actin or GAPDH. Full scans of western blot assays are shown in Additional file 1: Figs. S1–S4.

### Data and statistical analysis

All data displayed in the text and figures are expressed as mean ± SEM. Statistical differences between two groups were analyzed using the unpaired student’s t test, and statistical differences between multiple groups were analyzed using the one-way analysis of variance (ANOVA) using a SPSS statistical software (version 26.0, SPSS; IBM, Armonk, NY, USA). Statistical significance was determined with *p* value < 0.05.

## Results

### Effects of mangiferin (MGF) and T0901317 (T0) on inhibition of AS progression

To examine whether the combined treatment of MGF and T0 exerts hypolipidemic effects in atherosclerotic plaque formation without fatty liver and hyperglyceridemia, *Apoe*^*−/−*^ mice fed with HFD were administrated with T0, MGF or both T0 and MGF by gavage for 16 weeks. During the course of treatment, we daily checked body weight and intake of food and water, and there was no differences between control group and every treatment group.

**Fig. 1:**
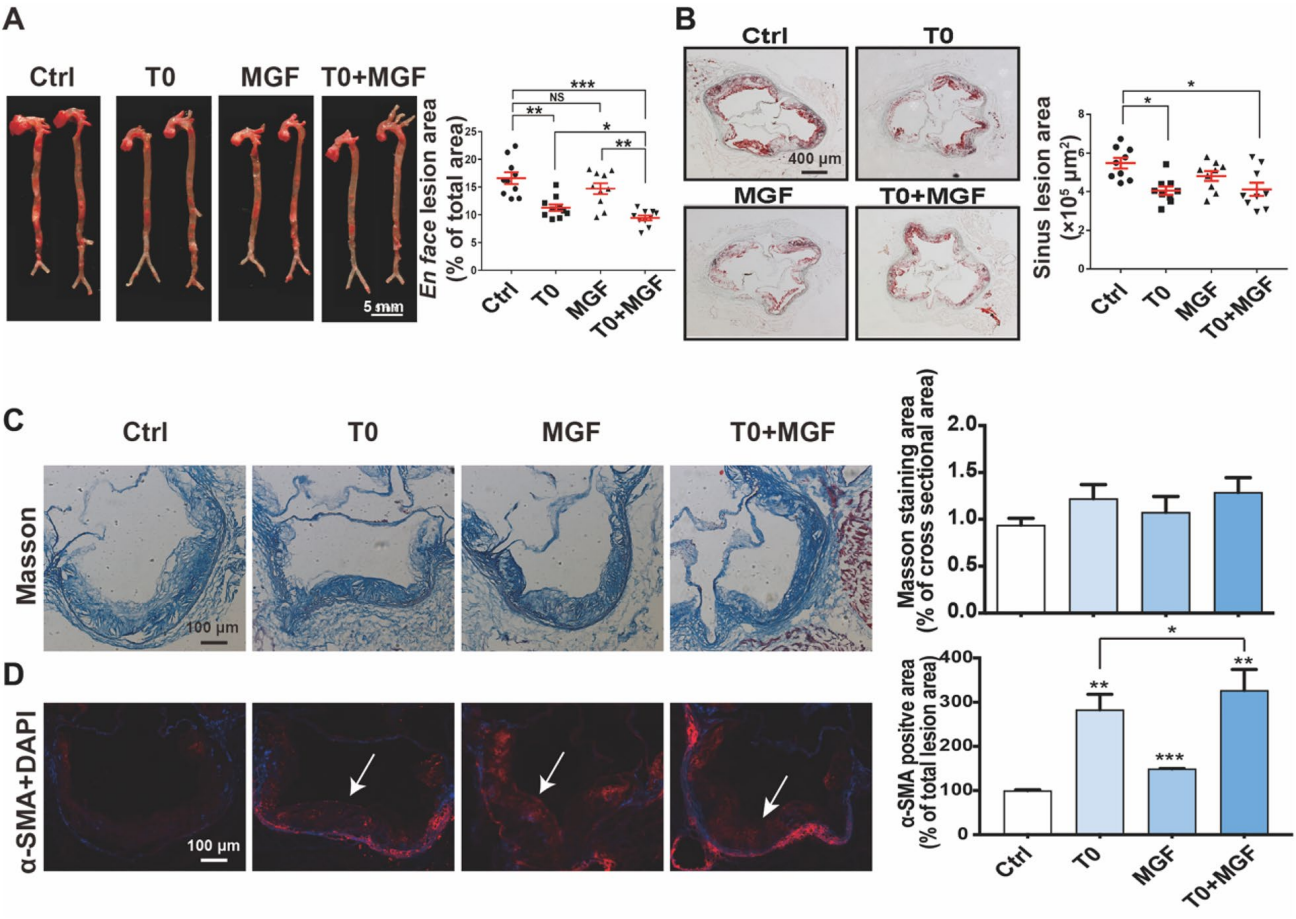
Effects of mangiferin (MGF) and T0901317 (T0) on inhibition of AS progression. *Apoe*^*−/−*^ mice fed with high fat diet (HFD) in four groups (n = 10/group) received the following treatment for 16 weeks. Ctrl: vehicle; T0: oral gavage of T0 (1 mg∙kg^−1^ bodyweight/day); MGF: oral gavage of MGF (200 mg kg^−1^ bodyweight/day); T0 + MGF: oral gavage of T0 and MGF. After treatment, aortas were collected for the following assays. **A** Lesions in *en face* aorta were determined by Oil Red O staining and lesion areas were expressed as % of total *en face* aorta areas. Scale bar, 5 mm. ^***^p < 0.001, ^**^p < 0.01, ^*^p < 0.05, NS: not significant (n = 10). **B** Sinus lesions in aortic root were determined by Oil Red O staining and lesion areas were expressed as μm^2^/section. Scale bar, 400 μm. ^*^p < 0.05 (n = 9). **C** Collagen content areas were determined by Masson staining of aortic root cross sections and quantified by a computer-assisted image analysis protocol (n = 3). Scale bar, 100 μm. **D** Expression of α-SMA (stained with red fluorescent color) was determined by immunofluorescent staining of aortic root cross sections and quantified by a computer-assisted image analysis protocol. Scale bar, 100 μm. ^***^p < 0.001, ^**^p < 0.01, ^*^p < 0.05 (n = 3)

At the end of experiment, we assessed the aortic lesions. Compared with control group, the *en face* aortic lesions in mice receiving T0 or MGF alone were inhibited by 33 and 20% respectively (Fig. 1A). The lesions were more inhibited by 47% after combined treatment of T0 and MGF, showing that MGF improved the inhibition of AS activated by LXR agonist. Similarly, T0 alone obviously inhibited sinus lesions in aortic root, and combined treatment maintained the efficacy of T0 (Fig. 1B).

Collagen content areas demonstrate stabilization of lesion plaques and suppression of plaque rupture [21]. Masson staining of aortic root cross section exhibited increasing positive staining areas tendency by the treatments (Fig. 1C). Further red immunofluorescent staining with antibody to α-SMA (the specific marker for vascular smooth muscle cells, representing stabilization of lesion plaques), exhibited T0 alone enhanced α-SMA in most lesion areas, and MGF alone enhanced α-SMA expression in the cap areas of lesions significantly (Fig. 1D). The combined treatment increased α-SMA expression more greatly. Taken together, the data in Fig. 1 clarifies that the combination of T0 and MGF could enhance the inhibition of AS progression by T0 without any inhibitory effects.

### Treatment of T0 and MGF activates expression of LXR target molecules in macrophage and inhibits foam cell formation

**Fig. 2:**
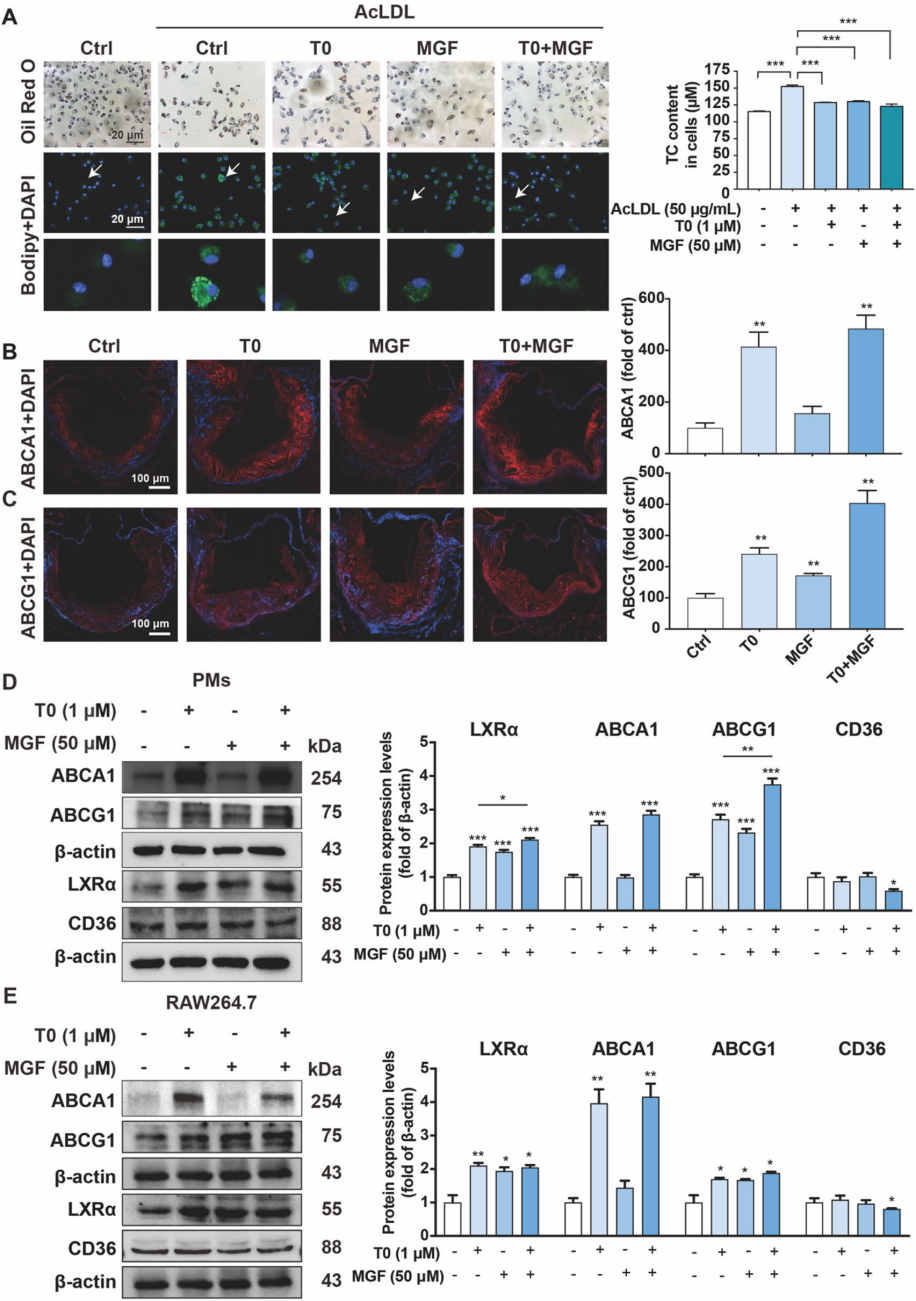
Treatment of T0 and MGF activates expression of LXR target molecules in macrophage and inhibits foam cell formation. **A** Peritoneal macrophages collected from mice were stained with Oil Red O or anti-bodipy antibody (green) to evaluate formation of foam cells (> 10 lipid droplets per cell, > 10 fields per sample), scale bar, 20 μm; and cholesterol contents were measured. ^***^p < 0.001, n = 3. Expression of (**B**) ABCA1 and (**C**) ABCG1 (stained with red fluorescent color) was determined by immunofluorescent staining of aortic root cross sections from *Apoe*^*−/−*^ mice and quantified by a computer-assisted image analysis protocol. Scale bar, 100 μm. ^**^p < 0.01, n = 3. Expression of ABCA1, ABCG1, LXRα and CD36 in cultured peritoneal macrophages (**D**) and RAW264.7 cells (**E**) was determined by western blot with total proteins extracted from cell samples. ^***^p < 0.001, ^**^p < 0.01, ^*^p < 0.05 (n = 3)

Since the imbalance of modified low density lipoprotein (LDL) internalization leading to foam cell formation [22], we next used acetylated LDL (AcLDL) to enhance intracellular cholesterol concentration in the macrophage. Primary peritoneal macrophages were incubated with AcLDL for 5 h to induce lipid accumulation leading to foam cell formation, which was confirmed by Oil Red O staining. Treatment with T0 or MGF alone decreased the differentiated foam cells, and combined treatment could increase the reducing tendency (Fig. 2A). Similarly, as expected, incubation of primary peritoneal macrophages with AcLDL contributed to the accumulation of the green fluorescent bodipy-positive lipid droplets. Treatment with T0 and MGF further reduced bodipy-positive lipid droplets (Fig. 2A). Consistently, the measurement of cholesterol contents within the macrophage showed combined treatment of T0 and MGF obviously decreased foam cells loaded cholesterol (Fig. 2A).

To assess the inhibitory effects of foam cell formation in lesion area on expression of the transporters ABCA1 or ABCG1, aortic root cross sections were stained with red fluorescent antibodies against ABCA1 or ABCG1 by immunofluorescent staining. Expressions of ABCA1 and ABCG1 were enhanced by T0 alone treatment, whereas, MGF alone slightly induced ABCG1 expression without ABCA1. Combined treatment of T0 and MGF showed significant stimulation in the expression of both ABCA1 and ABCG1 (Fig. 2B, C).

The effects of T0 and MGF on expression of ABCA1 and ABCG1 were evaluated by in vitro experiments, showing that treatment of T0 and MGF activated expression of LXRα and its targeted molecules ABCA1 and ABCG1 in primary peritoneal macrophages and RAW264.7 cells (Fig. 2D, E). T0 alone increased macrophage LXRα, ABCA1 and ABCG1 expression, in contrast, MGF alone moderately enhanced LXRα and ABCG1 expression, but not ABCA1 expression. In addition, combined treatment of T0 and MGF further reduced expression of cell surface scavenger receptors CD36, not seen in the treatment of T0 or MGF alone.

### Autophagy is implicated in lipid droplet degradation under combined treatment of T0 and MGF

**Fig. 3:**
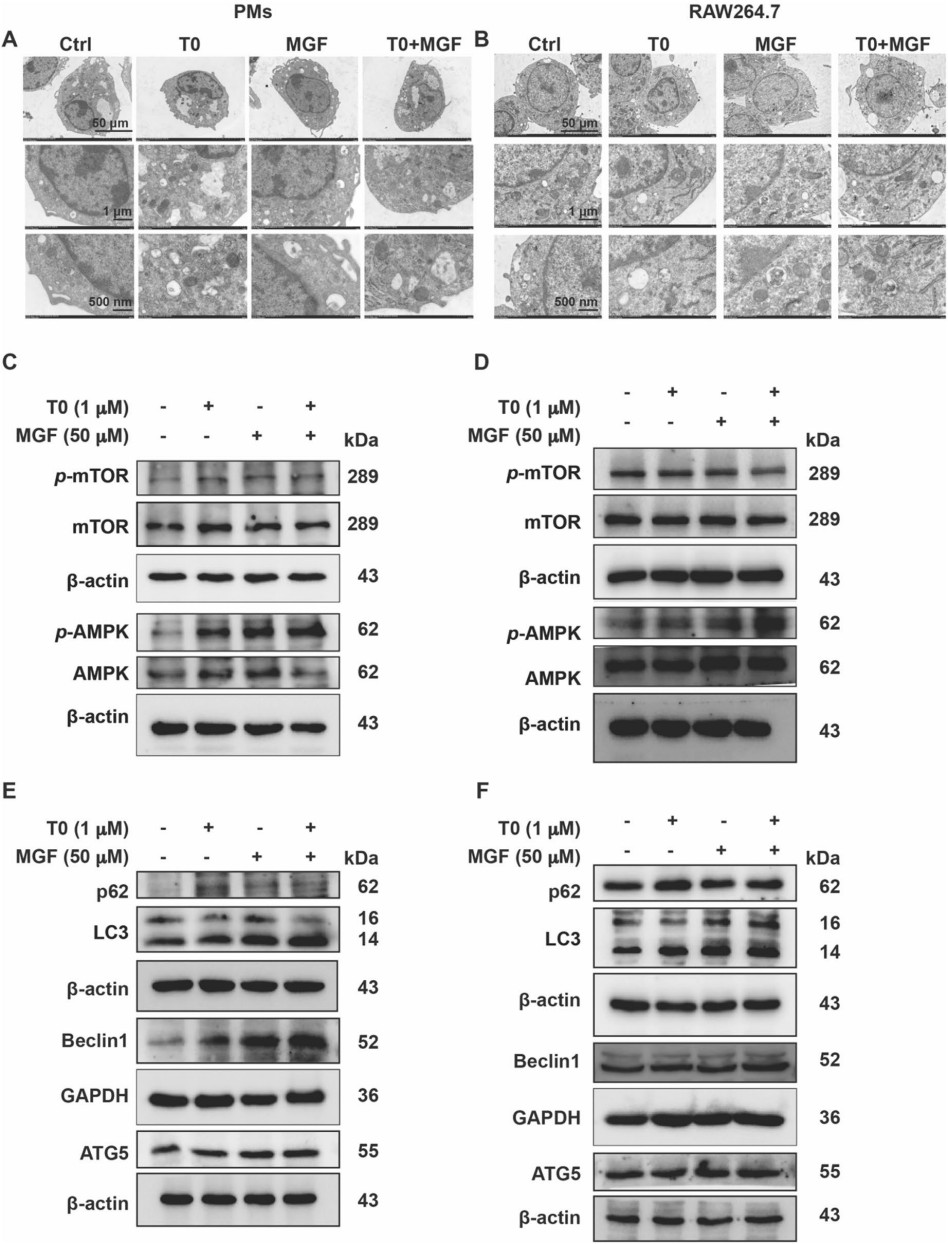
Autophagy is implicated in lipid droplet degradation under combined treatment of T0 and MGF. Representative digital images of electron microscopy reveal autophagic vacuoles accumulating in the cytoplasm of peritoneal macrophages (**A**) and RAW264.7 cells (**B**). Scale bar, 50 μm, 1 μm, 500 nm. Expression of *p*-mTOR, mTOR, *p*-AMPK, and AMPK in peritoneal macrophages (**C**) and RAW264.7 cells (**D**) was determined by western blot with total proteins extracted from cell samples. Expression of p62, LC3, Beclin1 and ATG5 in peritoneal macrophages (**E**) and RAW264.7 cells (**F**) was determined by western blot with total proteins extracted from cell samples

Lipid-laden macrophages, or foam cells, are a hallmark of AS by the buildup of cholesterol-rich plaques in the artery wall [23]. Within macrophage foam cells, lipid droplet catabolism via autophagy release free cholesterol for efflux, a process dependent on ABCA1 and ABCG1. Electron microscopy showed that complete double-membrane autophagosome were accumulated in T0 and MGF treated peritoneal macrophages (Fig. 3A) and RAW264.7 cells (Fig. 3B).

A large body of evidence demonstrated that intracellular cholesterol depletion also initiates autophagy, a process of particular relevance to AS [24]. Regulation of autophagy contains mTOR as an upstream signal [25]. To investigate whether mTOR-mediated signaling is involved in combined treatment of T0 and MGF, the phosphorylation of mTOR and AMPK was analyzed, showing that T0 or/and MGF treatment promoted phosphorylation of mTOR and AMPK (Fig. 3C, D). As a consequence, autophagy classical marker proteins, such as p62, LC3, beclin1 and ATG5, were consistently increased by the treatment of T0 or/and MGF (Fig. 3E, F). Notably, either T0 or MGF treatment employs macrophage autophagy to undergo lipid degradation.

### Autophagic flux modulates foam cell cholesterol efflux under combined treatment of T0 and MGF

**Fig. 4:**
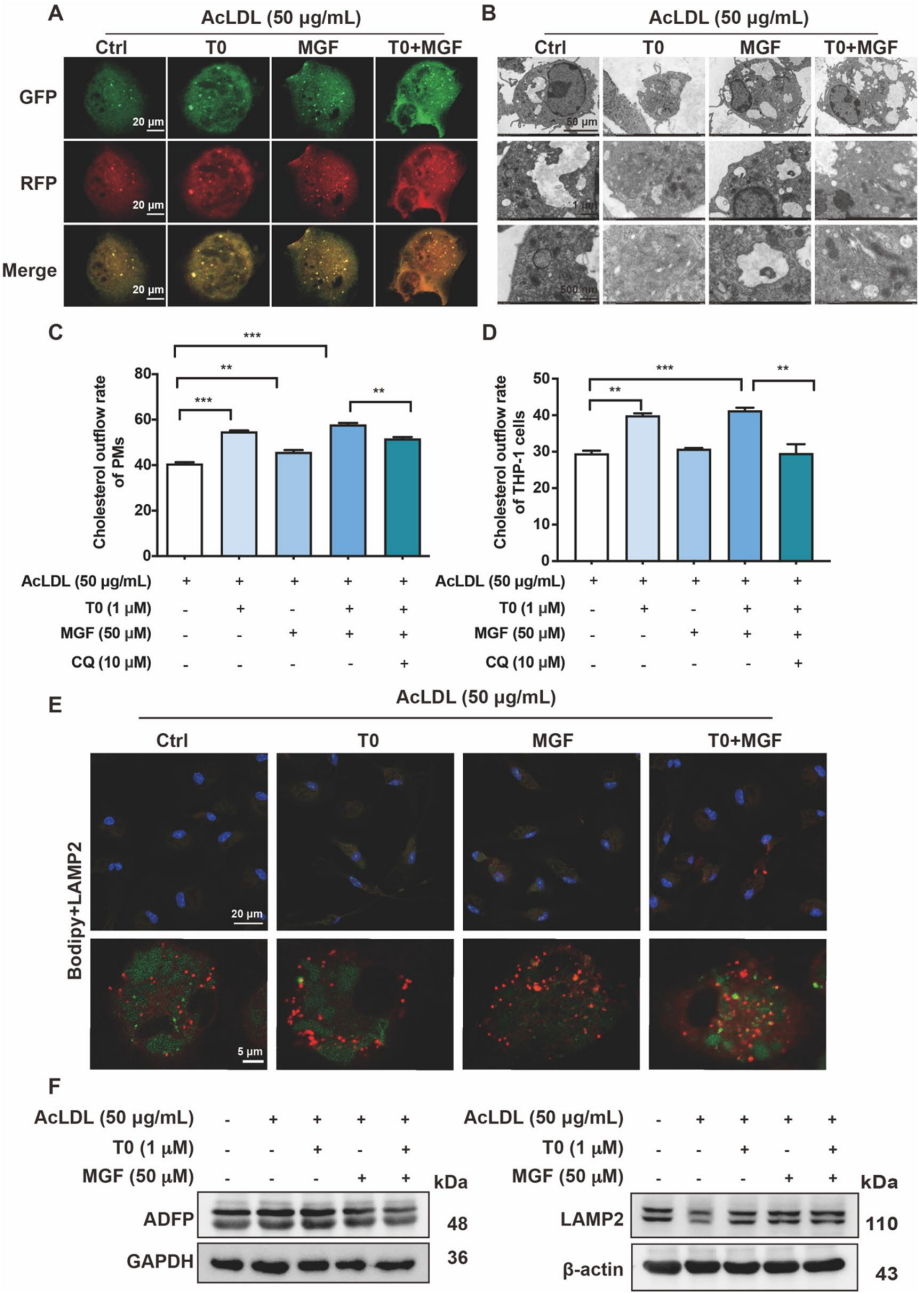
Autophagic flux modulates foam cell cholesterol efflux under combined treatment of T0 and MGF. **A** Peritoneal macrophages incubated with AcLDL were assayed for the puncta of GFP-LC3 (green)/RFP-LC3 (red) during the early stages of autophagy. Scale bar, 20 μm. **B** Representative digital images of electron microscopy reveal autophagic vacuoles accumulating in the cytoplasm of peritoneal macrophages treated with AcLDL. Scale bar, 50 μm, 1 μm, 500 nm. Quantitation of cholesterol outflow rate with or without chloroquine (CQ) under combined treatment of T0 and MGF in peritoneal macrophages (**C**) and human THP-1 macrophage treated with AcLDL (**D**). **E** Peritoneal macrophages were stained with bodipy (green) and LAMP2 (red) to identify lipophagy in macrophage foam cells under AcLDL-treated conditions. Scale bar, 20 μm, 5 μm. **F** Expression of ADFP and LAMP2 in AcLDL-treated peritoneal macrophages was determined by western blot with total proteins extracted from cell samples

To further verify the effect of T0 and MGF combined treatment on lipid droplet degradation through autophagic flux in macrophages, autophagy signaling pathways were optimized in AcLDL-treated macrophages. Peritoneal macrophages carrying either the gene for the green fluorescent protein (GFP)-MAP1LC3B/LC3B (microtubule-associated protein 1 light chain 3β) or the gene for the red fluorescent protein (RFP)-LC3B, were assayed for the puncta of GFP-LC3/RFP-LC3 during the early stages of autophagy. Compared to the control, T0 and MGF treatment displayed a more punctate pattern of fluorescence under AcLDL-treated conditions (Fig. 4A). Meanwhile, electron microscopy showed that complete double-membrane autophagosome were accumulated in T0 and MGF treated peritoneal macrophages under AcLDL-treated conditions to enhance lipophagy (Fig. 4B).

To confirm quantitative differences in cholesterol efflux in autophagy suppression, we next measured cholesterol outflow rate with or without chloroquine (CQ) under combined treatment of T0 and MGF in peritoneal macrophages (Fig. 4C) and human THP-1 macrophages (Fig. 4D). Obviously, autophagy deficiency impairs foam cell cholesterol efflux under combined treatment of T0 and MGF. Next, peritoneal macrophages were incubated with green fluorescent bodipy (a commonly used fluorescent neutral-lipid dye) and stained with red fluorescent lysosome-associated membrane protein type 2 (LAMP2) to identify lipophagy in macrophage foam cells under AcLDL-treated conditions (Fig. 4E). Combined treatment of T0 and MGF significantly accelerates lipophagy to enhance lipid degradation, which is also confirmed by downregulation of adipocyte differentiation-related protein (ADFP) and upregulation of LAMP2 through western blot analysis (Fig. 4F).

### Treatment of T0 and MGF blocks LXR-induced fatty liver and hyperglyceridemia

**Fig. 5:**
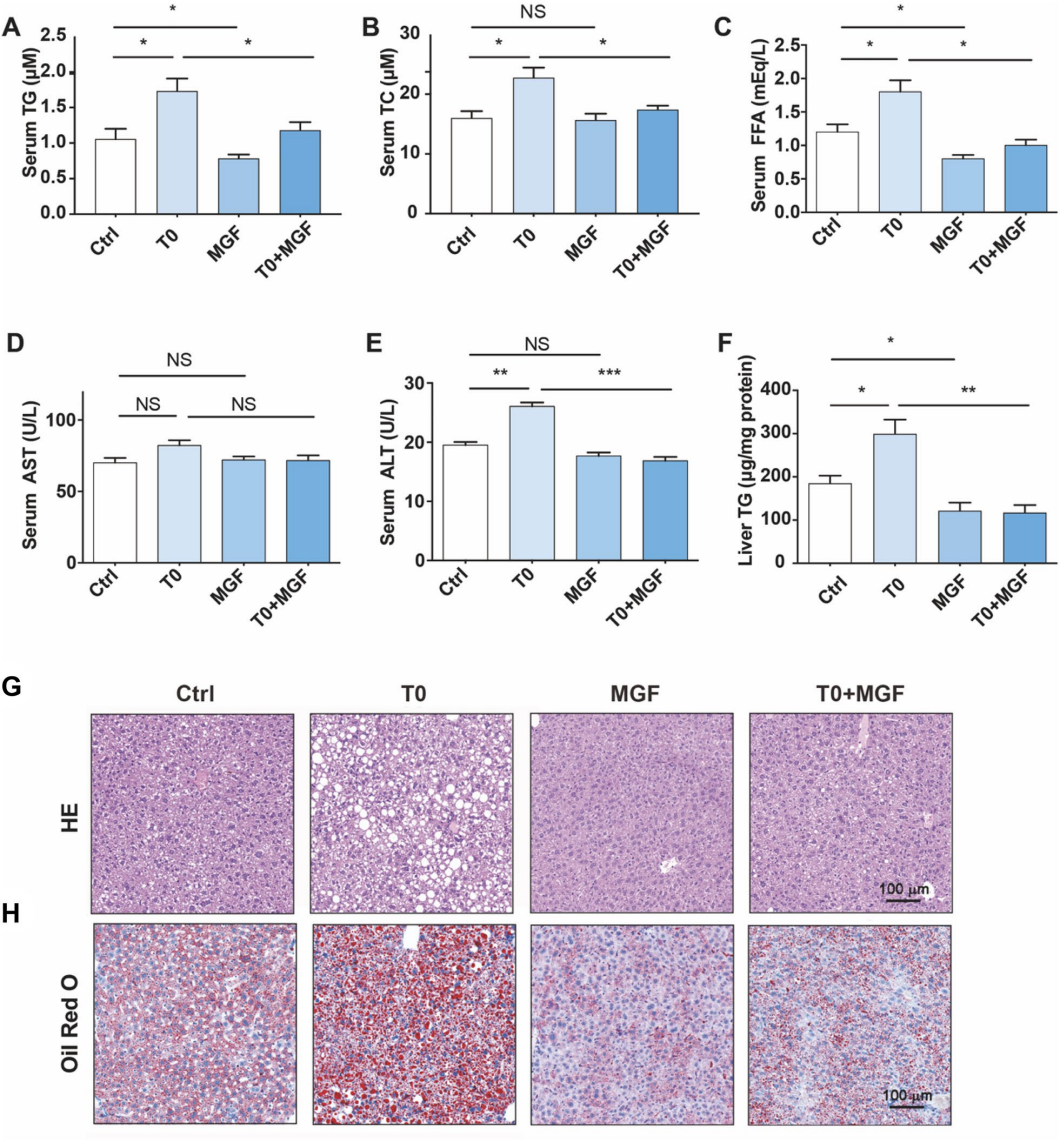
Treatment of T0 and MGF blocks LXR-induced fatty liver and hyperglyceridemia in *Apoe*^*−/−*^ mice. **A** Serum TG, (**B**) TC, (**C**) FFA, (**D**) AST and (**E**) ALT levels were analyzed by commercial kits. **F** Hepatic TG quantitative analysis with total liver lipid extract. ^***^p < 0.001, ^**^p < 0.01, ^*^p < 0.05 (n = 3). **G** HE attaining of liver paraffin sections. **H** Oil Red O staining of liver frozen sections. Scale bar, 100 μm. Ctrl: vehicle; T0: oral gavage of T0 (1 mg kg^−1^ bodyweight/day); MGF: oral gavage of MGF (200 mg∙kg^−1^ bodyweight/day); T0 + MGF: oral gavage of T0 and MGF

The most frequent side effect of T0 administration is fatty liver and high level of plasma TG, by activating LXRα and the transcription factor of lipogenic genes SREBP-1c [26]. Thus, quantitation of lipid levels in the serum of *Apoe*^*−/−*^ mice showed that T0 alone increased serum TG, TC, and FFA levels; however, this stimulation was consequently reduced by MGF additional treatment, not consistent with effect of MGF alone on serum TC level of *Apoe*^*−/−*^ mice (Fig. 5A–C). Level of serum ALT, rather than AST, was increased by T0 and this increase was also substantially attenuated by MGF (Fig. 5D, E). Quantitation of TG levels in the liver showed that T0 elevated hepatic TG levels and that MGF significantly reduced hepatic TG accumulation of *Apoe*^*−/−*^ mice with or without T0 administration (Fig. 5F). HE and Oil Red O staining also confirmed that T0 alone aggravated hepatic vacuolation and lipid abnormal accumulation, and this increase was attenuated by MGF (Fig. 5G, H).

### Treatment of T0 and MGF activates AMPK signaling pathway to suppress hepatic lipogenesis and induce autophagy to accelerate hepatic degradation

**Fig. 6:**
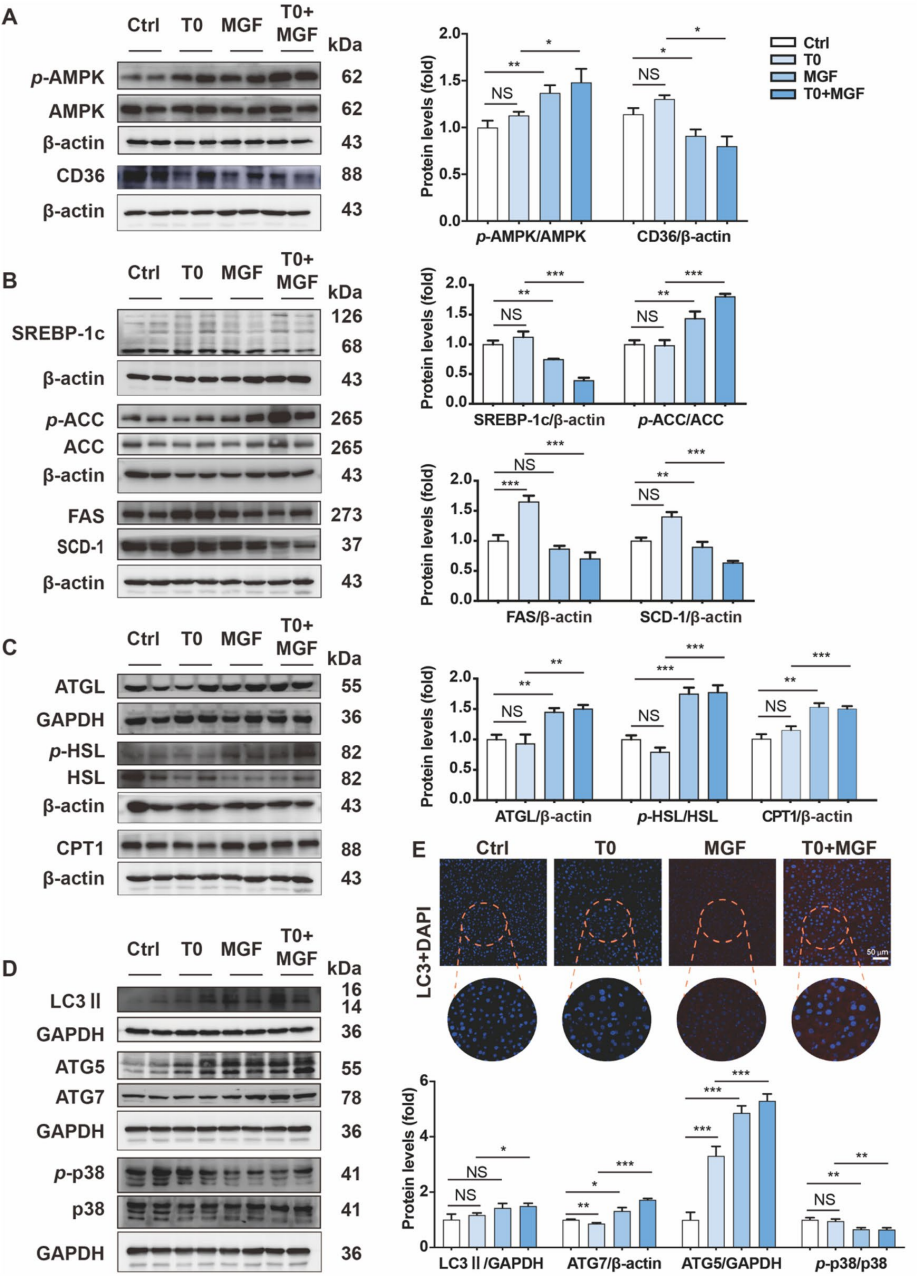
Treatment of T0 and MGF activates AMPK signaling pathway to suppress hepatic lipogenesis and induce autophagy to accelerate hepatic degradation in *Apoe*^*−/−*^ mice. **A** Expression of *p*-AMPK, AMPK, and CD36 was determined by western blot with total proteins extracted from liver samples of *Apoe*^*−/−*^ mice. **B** Expression of lipid biosynthesis related SREBP-1c, *p*-ACC, ACC, FAS and SCD-1. **C** Expression of lipolysis related ATGL, *p*-HSL and HSL, and β-oxidation relative CPT1. **D** Expression of autophagy related LC3II, ATG5, ATG7, *p*-p38 and p38. ^***^p < 0.001, ^**^p < 0.01, ^*^p < 0.05 (n = 3). **E** Hepatic red fluorescent LC3 protein was determined by immunofluorescent staining. Scale bar, 50 μm

We further evaluated how MGF-activated signaling cascade regulated the hepatic lipid biosynthesis and lipolysis. Activation of AMPK in the presence of MGF was observed with or without T0 treatment in the liver of *Apoe*^*−/−*^ mice. Downregulation of cell surface scavenger receptors CD36 under MGF treatment reflected transportation of lipid droplets from blood circulation to liver was suppressed either (Fig. 6A). Abundant evidence clarifies that hepatic stimulating AMPK phosphorylation inhibits lipid biosynthesis and enhances FFA β-oxidation. Therefore, we confirmed that MGF significantly decreased protein expression of SREBP-1c, FAS and SCD-1, and increased phosphorylation of ACC in deed (Fig. 6B). Consistently, the lipolysis relative ATGL protein expression and HSL phosphorylation were enhanced to accelerate FFA β-oxidation through upregulation of CPT1 (Fig. 6C). Accumulating research shows that autophagy maintains lipid droplets degradation via hepatic lipophagy [27]. Our results found that MGF treatment stimulated protein expression of LC3II, ATG5 and ATG7, in consistent with downregulating p38 phosphorylation under T0 treatment (Fig. 6D). Similarly, hepatic red fluorescent LC3 protein determined by immunofluorescent staining exhibited MGF enhanced autophagy to accelerate lipolysis for FFA β-oxidation (Fig. 6E).

## Discussion

**Fig. 7:**
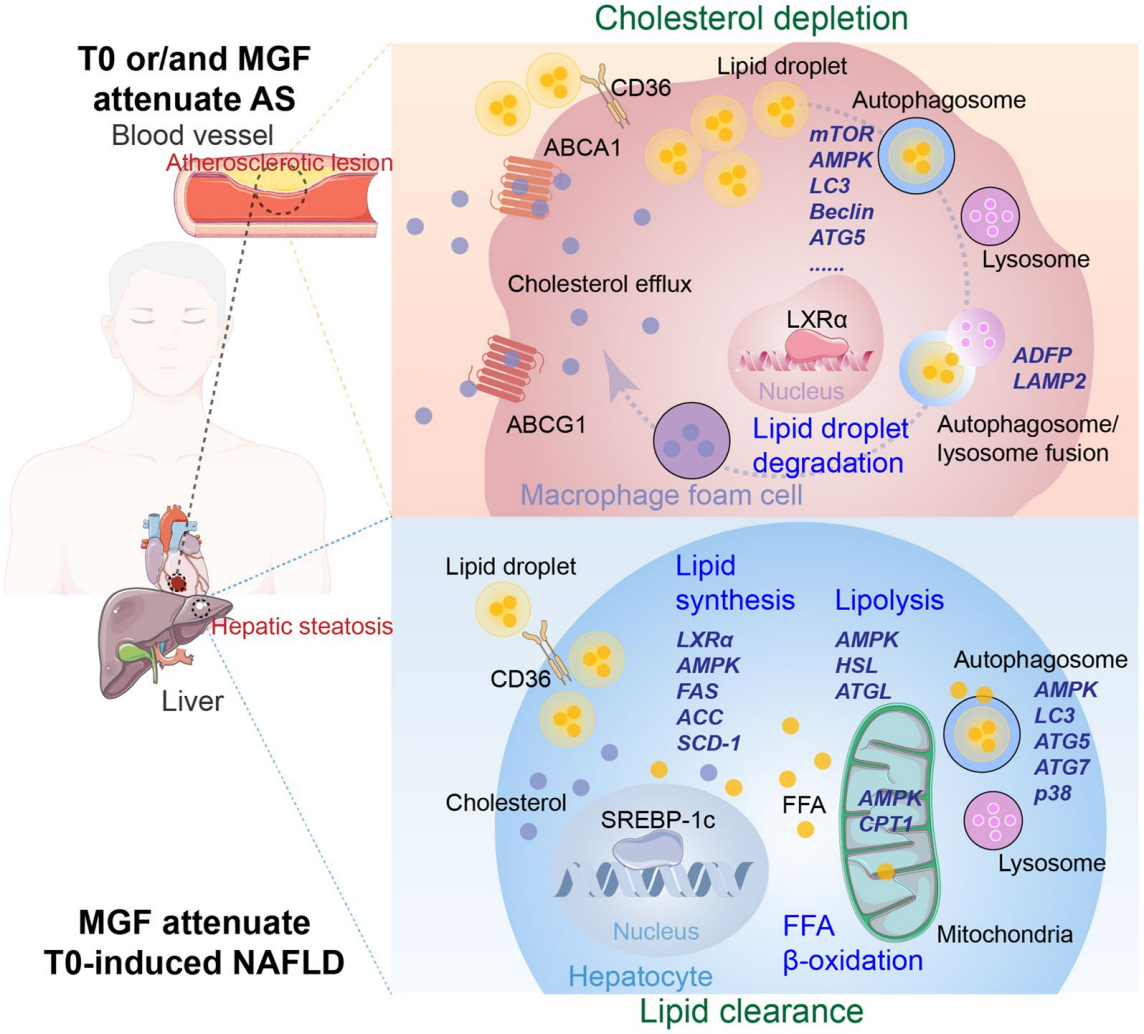
Model for the function of combined treatment of T0 and MGF in the AS related dyslipidemia targeting autophagy in cholesterol efflux from macrophage foam cells. On the one hand, T0 alone or T0 and MGF promote macrophage cholesterol efflux by activating LXRα to augment the expression of ABCA1 and ABCG1 and also enhance lipophagy in atherosclerotic lesion. Of note, in mammalian macrophage foam cells, lipid droplets are tagged for autophagic fusion, possibly beginning with mTOR-AMPK signaling to initiate lipid droplet degradation for further cholesterol depletion. On the other hand, T0-induced hepatic lipid abnormal accumulation is attenuated by MGF. In this scenario, MGF may activate AMPK signaling to suppress lipid synthesis and accelerate lipolysis for β-oxidation of free fatty acid. Alternatively, hepatic lipid droplets may be degraded through autophagy mechanism mediated by AMPK, and relative classical factors LC3, ATGs and p38 to facilitate hepatic lipophagy

The synthetic LXR ligand T0 reduced AS but activated hepatic SREBP-1c mediated lipogenesis. Aa a result, the drawbacks slowed down the progression of LXR agonists into the clinic and hindered the development of multiple promising LXR products after Phase I clinical trials [28]. Optimizing the drug delivery or combination therapy of an LXR agonist to macrophages in atherosclerotic plaques without hepatic side effects represents a fundamentally new approach to treat AS. Attentions to synthetic LXR ligand T0 for AS therapy have developed innovative nano-sized drug delivery to attenuate AS without side effect on hepatic lipogenesis, such as nanofiber hydrogel D-Nap-GFFY-T0901317 [29, 30] or T0-synthesis HDL nanoparticles [28, 31]. Moreover, the combination of T0 and metformin also prompts that addition of metformin to T0 can stimulate T0 effects on reducing AS with blocking T0-induced hepatic lipogenesis [18]. As the same way, the present study optimized effects of T0 on AS with no side effects through the combination of MGF derived from *Mangifera indica* L. in *Apoe*^*−/−*^ mice. The results confirmed great protection of MGF and T0 administration against atherosclerotic lesions and restored *Apoe*^*−/−*^ mice hepatic hyperglycerides substantially. We further put forward three main signaling pathways in which MGF and T0 promotes LXRα and mTOR/AMPK signaling cascade in macrophage; and promotes AMPK signaling cascade in hepatocyte, leading to lipid metabolic homeostasis (Fig. 7). This study thus confers MGF as a potential assistant T0-cotreatment to AS.

In *Apoe*^*−/−*^ mice, expressions of ABCA1 and ABCG1 in aortic root cross sections were enhanced by T0 alone treatment, whereas, MGF alone slightly induced ABCG1 expression without ABCA1. Combined treatment of T0 and MGF showed significant stimulation in the expression of both ABCA1 and ABCG1. Furthermore, ABCA1 and ABCG1 expressions are increased in response to LXRα activation by T0 in primary peritoneal macrophages and RAW264.7 cells. However, the difference between MGF alone and MGF and T0 combination is not consistently significant in several anti-atherogenic actions, suggesting the efficiency of MGF on macrophage ABCG1 expression was independent of the LXR pathway. Additionally, T0 and MGF treatment further reduced expression of CD36 to inhibit cholesterol input from cell membrane. We next identified autophagy promoted by MGF and T0 treatment establishes a positive feedback loop that increases cholesterol efflux, resulted from LXRα activation. It is pointed out that MGF and T0 stimulated mTOR/AMPK signaling cascade to initiate autophagy, shown as complete double-membrane autophagosome in T0 and MGF treated peritoneal macrophages and RAW264.7 cells through electron microscopy.

Autophagy itself is linked to lysosomal cholesterol pools. We conclude an important role for lysosomes in lipid droplets degradation in macrophage foam cells under MGF and T0 treatment. Additionally, we demonstrate that lipid droplets are delivered to lysosomes through autophagy, where free cholesterols are generated to form efflux from macrophages, a significant step in reverse cholesterol transport, which is fundamental to the regression of atherosclerotic plaques [32]. There are many models for foam cell formation in vitro, such as incubation of macrophages with atherogenic lipoproteins, including AcLDL, OxLDL, AgLDL, and VLDL [8, 33]. Multiple pieces of evidence support that lipid droplets are processed in lysosomes in the AcLDL-loaded macrophages in our study. We used Oil Red O and bodipy staining to examine lipids location and reduction after MGF and T0 treatment. Thus, the decrease in cellular cholesterol contents and cholesterol outflow rate was obvious in the presence of MGF and T0. Furthermore, using an autophagy inhibitor CQ during AcLDL uptake showed increasing cholesterol outflow rate was nearly abolished. Finally, we analyzed the downregulation of ADFP and upregulation of LAMP2 under MGF and T0 treatment, which confirmed the combination accelerated lipophagy to enhance cholesterol efflux in macrophage after AcLDL loading.

Mechanically, free LXR ligand induces hyperglyceridemia and liver steatosis through activating SREBP-1c cascades reaction due to LXRα activation. Thus, activation of LXRβ or application of tissue-specific ligand may be suitable for AS therapy. Indeed, LXRβ agonists, such as GW6340, was reported little effect on hepatic lipogenic genes [34]. The agonist LXR-623 (WAY 252623) was confirmed also effective in reverse cholesterol transport in macrophage without hepatic side effects. Whereas, the Phase I clinical trial of LXR-623 was terminated due to its central nervous system adverse effects [35]. Under the consideration of the similarity of the ligand binding domain between LXRα and LXRβ, investigation of combination therapy was performed, which used LXR ligand and another chemical, to sustain inhibiting formation of atherosclerotic plaques and avoid hepatic lipogenesis. Previous studies have investigated that combination therapy of LXR-623 and simvastatin [36], or T0 and MEK1/2 inhibitor (U0126) [37], showing plaque regression with limited lipogenesis. Moreover, metformin was also clarified effective in benefiting T0-induced AS and attenuating T0-induced lipogenesis. Our previous study found that MGF prevented hepatic lipid metabolic disorders via SIRT-1/AMPK pathway [14]. So we further examined the combined effects of T0 and MGF on AS and T0-induced lipogenesis. As a consequence, high expression of lipogenic genes and development of fatty liver triggered by LXRα activator T0 was dramatically attenuated by combination with MGF. Additionally, MGF was further reported as the main polyphenol compound to protect against AS-prone hypercholesterolemic mouse through antioxidant capacity [38]. In this study, we confirmed AMPK activation of MGF in the hepatocyte, contributing to suppressing lipogenesis and enhancing lipid oxidation. Next, we also demonstrated that autophagosome clearance by lysosomes was also processed by MGF under hepatic lipogenic condition in *Apoe*^*−/−*^ mice.

In summary, we provide several lines of evidence that combination of MGF and T0 prevents atherosclerotic plaque progression in atherogenic mouse models, and reduce cholesterol content in the macrophage foam cells, targeting autophagy-dependent macrophage cholesterol efflux. Furthermore, MGF attenuates fatty liver induced by T0 targeting AMPK signaling cascade also in the presence of autophagy in the hepatocyte of *Apoe*^*−/−*^ mice. Although further studies are required to clarify the relation between dosages and effects of MGF and T0 in the regulation of autophagy in macrophages and AS relative dyslipidemia, our results suggest that combination of MGF and T0 may provide therapeutic potential to enhance macrophage cholesterol efflux without fatty liver in a autophagy dependent manner.

## Conclusion

Altogether, our findings reveal that MGF and T0 engages in AS therapy without side effects by activating AMPK-dependent autophagy to promote macrophage cholesterol efflux, and MGF might serve as a natural compound to assist T0 in AS via targeting autophagy.

### Supplementary Information

**Additional file 1: Fig S1.** Full scans of western-blot data were shown in Fig. 2. Rectangles delimit cropped areas used in the indicated panels in Fig. 2. β-actin was used as an internal control. **Fig. S2.** Full scans of western-blot data were shown in Fig. 3. Rectangles delimit cropped areas used in the indicated panels in Fig. 3. β-actin or GAPDH was used as an internal control. The protein expression statistical analysis was shown in histogram. Data are expressed as means ± SEM (n = 3). ^***^p<0.001, ^**^p<0.01, ^*^p<0.05 (n=3). **Fig S3.** Full scans of western-blot data were shown in Fig. 4. Rectangles delimit cropped areas used in the indicated panels in Fig. 4. β-actin or GAPDH was used as an internal control. The protein expression statistical analysis was shown in histogram. Data are expressed as means ± SEM (n = 3). ^***^p<0.001, ^**^p<0.01, ^*^p<0.05 (n=3). **Fig S4.** Full scans of western-blot data were shown in Fig. 6. Rectangles delimit cropped areas used in the indicated panels in Fig. 6. β-actin or GAPDH was used as an internal control.

## Data Availability

The datasets used and/or analysed during the current study are available from the corresponding author on reasonable request.

## Abbreviations

AS: Atherosclerosis
ABCA1: ATP-binding cassette transporters from subfamilies A1
ABCG1: ATP-binding cassette transporters from subfamilies G1
ALT: Alanine aminotransferase
AST: Aspartate aminotransferase
LXR: Liver X receptor
T0: T0901317
SREBP-1c: Sterol regulatory element binding transcription factor 1c
MGF: Mangiferin
TG: Triglyceride
FFA: Free fatty acid
SIRT-1: Sirtuin-1
AMPK: AMP-activated protein kinase
α-SMA: Smooth muscle α-actin
HE: Hematoxylin–eosin
AcLDL: Acetylated low density lipoprotein
DAPI: 4’,6-Duamidino-2-phenylindole
TEM: Transmission electron microscopy
LDL: Low density lipoprotein
GFP: Green fluorescent protein
RFP: Red fluorescent protein
LAMP2: Lysosome-associated membrane protein type 2
ADFP: Adipocyte differentiation-related protein
