# Supplementary material for: Combination of mangiferin and T0901317 targeting autophagy promotes cholesterol efflux from macrophage foam cell in atherosclerosis
Source: Chin Med. 2024 Jan 5;19:5. doi: 10.1186/s13020-023-00876-9 (PMC10770909; doi:10.1186/s13020-023-00876-9)

Additional file 1

for

Combination of mangiferin and T0901317 targeting autophagy promotes cholesterol efflux from macrophage foam cell in atherosclerosis

Qian Chen[1,†], Sijian Wang[1,†], Ruixia Bao[1], Dan Wang[1], Yuzheng Wu[1], Yi Zhang[1], Mengyang Liu[1,*] and Tao Wang[1,2,*]

[1*] State Key Laboratory of Component Based Chinese Medicine, Institute of Traditional Chinese Medicine, Tianjin University of Traditional Chinese Medicine, 10 Poyanghu Road, Tianjin, 301617, China.

[2] State Key Laboratory of Bioactive Substance and Function of Natural Medicines, Institute of Materia Medica, Chinese Academy of Medical Sciences and Peking Union Medical College, 1 Xian Nong Tan Street, Beijing, 100050, China.

[*] Correspondence: Mengyang Liu and Tao Wang. E-mails: liumengyang0212@tjutcm.edu.cn; wangtao@tjutcm.edu.cn;

[†]Qian Chen and Sijian Wang have contributed equally as first authors.

Figure legends for additional file 1

Additional file 1: Fig S1. Full scans of western-blot data were shown in Fig. 2. Rectangles delimit cropped areas used in the indicated panels in Fig. 2. β-actin was used as an internal control.

Additional file 1: Fig S2. Full scans of western-blot data were shown in Fig. 3. Rectangles delimit cropped areas used in the indicated panels in Fig. 3. β-actin or GAPDH was used as an internal control. The protein expression statistical analysis was shown in histogram. Data are expressed as means ± SEM (n = 3). $^{***}p<0.001$, $^{**}p<0.01$, $^{*}p<0.05$ (n=3).

Additional file 1: Fig S3.. Full scans of western-blot data were shown in Fig. 4. Rectangles delimit cropped areas used in the indicated panels in Fig. 4. β-actin or GAPDH was used as an internal control. The protein expression statistical analysis was shown in histogram. Data are expressed as means ± SEM (n = 3). $^{***}p<0.001$, $^{**}p<0.01$, $^{*}p<0.05$ (n=3).

Additional file 1: Fig S4. Full scans of western-blot data were shown in Fig. 6. Rectangles delimit cropped areas used in the indicated panels in Fig. 6. β-actin or GAPDH was used as an internal control.

Additional file 1: Fig S1.

Fig. 2

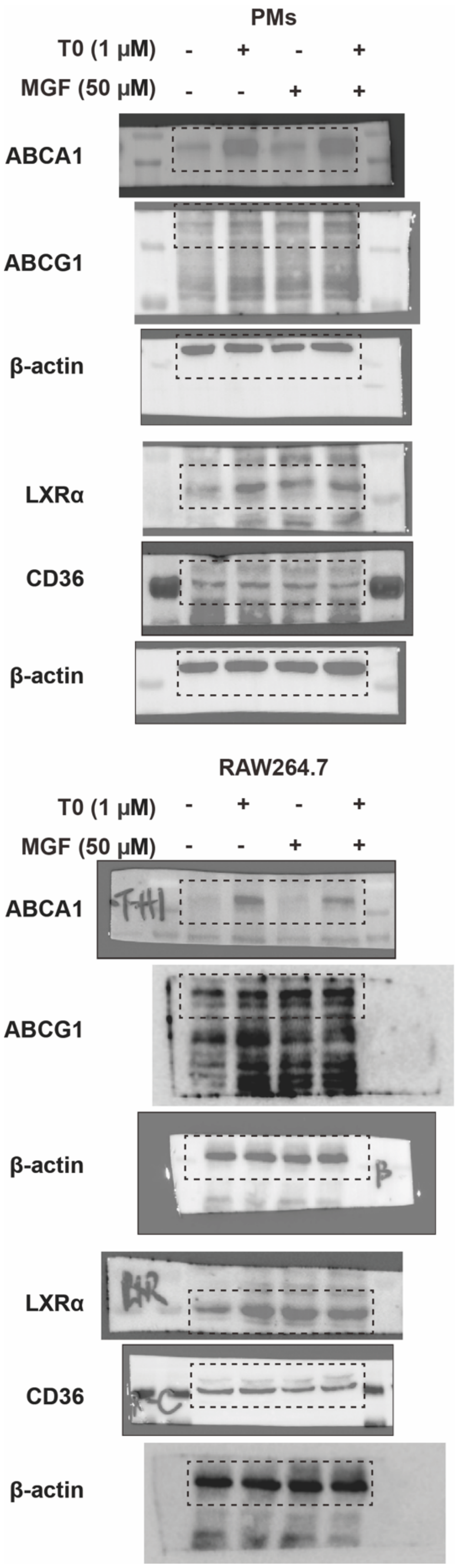

Additional file 1: Fig S2.

Fig. 3

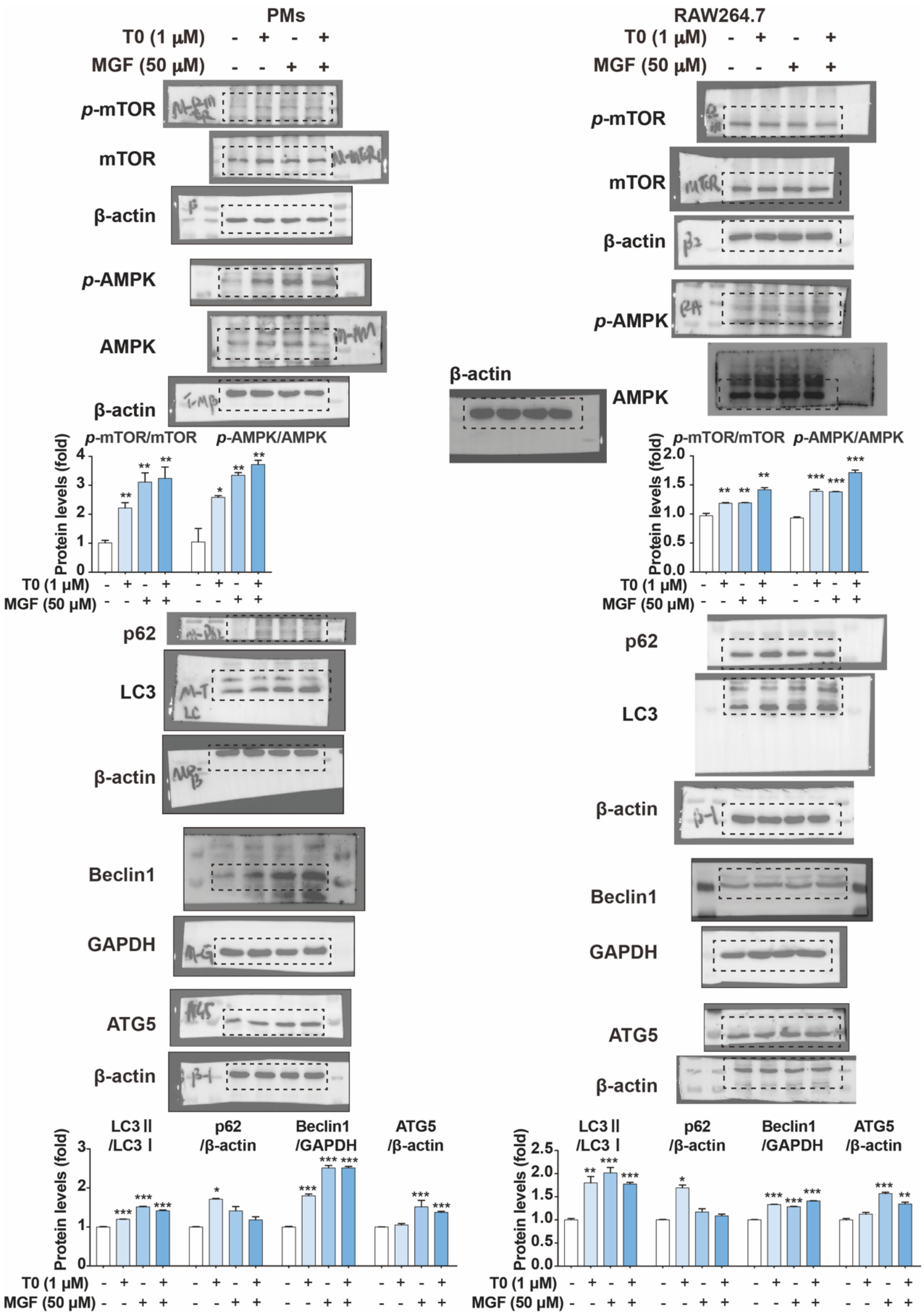

Additional file 1: Fig S3.

Fig. 4

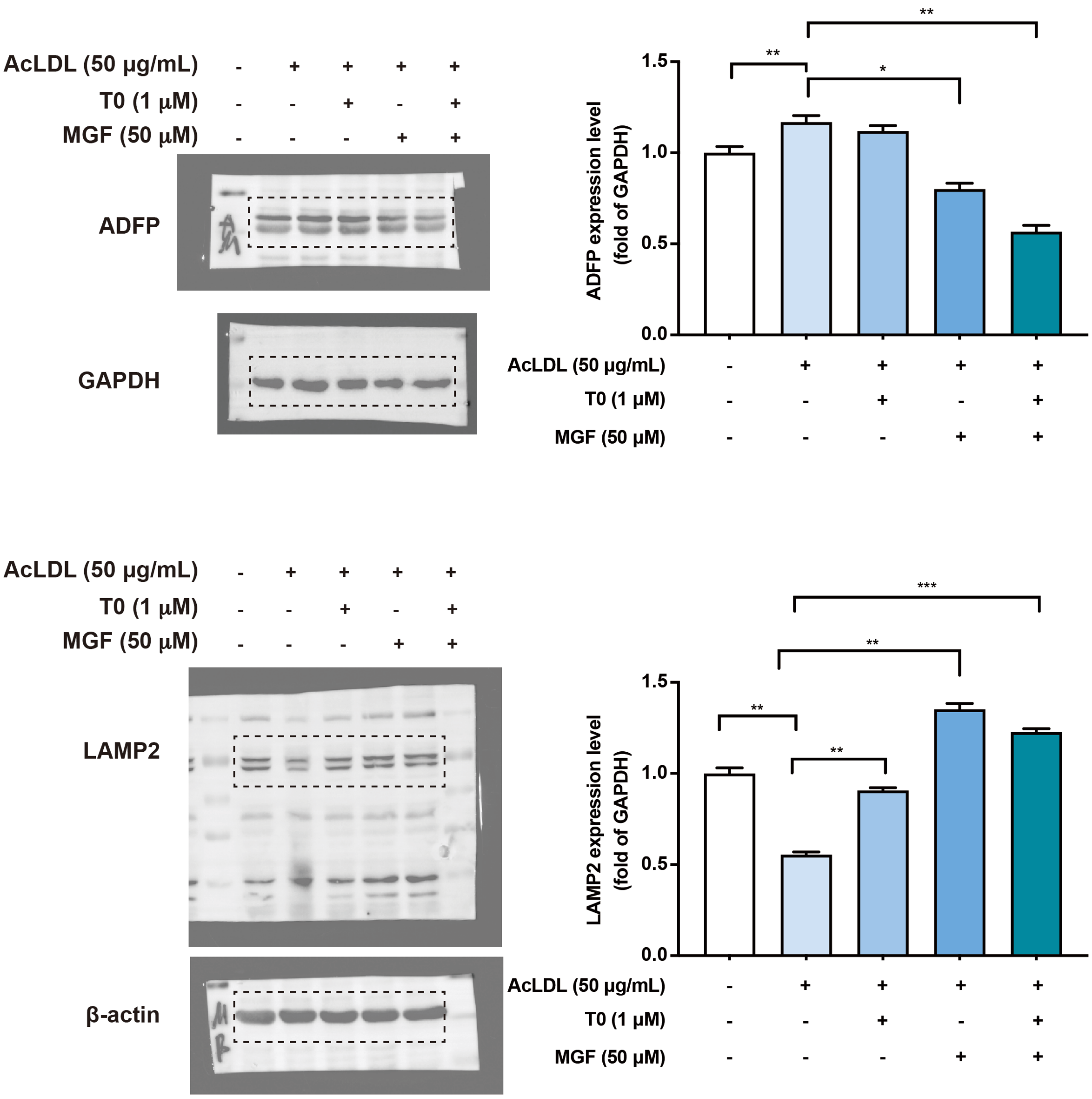

Additional file 1: Fig S4.

Fig. 6

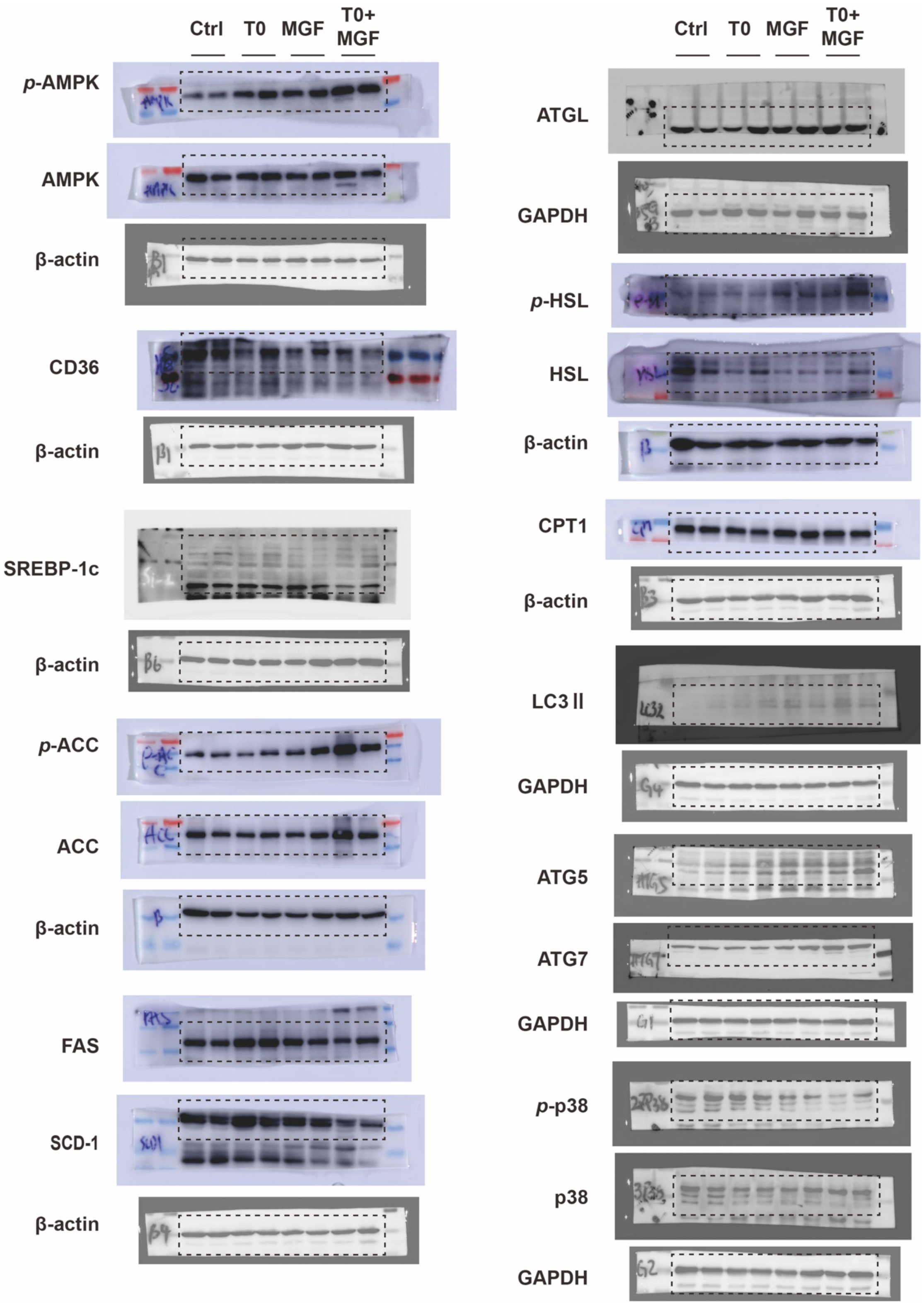

Supplement: Supplementary file 1 — Additional file 1: Fig S1. Full scans of western-blot data were shown in Fig. 2. Rectangles delimit cropped areas used in the indicated panels in Fig. 2. β-actin was used as an internal control. Fig. S2. Full scans of western-blot data were shown in Fig. 3. Rectangles delimit cropped areas used in the indicated panels in Fig. 3. β-actin or GAPDH was used as an internal control. The protein expression statistical analysis was shown in histogram. Data are expressed as means ± SEM (n = 3). ***p<0.001, **p<0.01, *p<0.05 (n=3). Fig S3. Full scans of western-blot data were shown in Fig. 4. Rectangles delimit cropped areas used in the indicated panels in Fig. 4. β-actin or GAPDH was used as an internal control. The protein expression statistical analysis was shown in histogram. Data are expressed as means ± SEM (n = 3). ***p<0.001, **p<0.01, *p<0.05 (n=3). Fig S4. Full scans of western-blot data were shown in Fig. 6. Rectangles delimit cropped areas used in the indicated panels in Fig. 6. β-actin or GAPDH was used as an internal control. [file 13020_2023_876_MOESM1_ESM.pdf]
